# Peripheral odontogenic keratocyst: Report of two new cases and review of the literature

**DOI:** 10.4317/jced.57653

**Published:** 2020-10-01

**Authors:** Bruno-Teixeira-Gonçalves Rodrigues, Mônica-Simões Israel, Kenia-Lorena-Monteiro de Moura, Giulianna-Lima Pinheiro, Roman Carlos, Fábio-Ramoa Pires

**Affiliations:** 1 DDS, Dental School, State University of Rio de Janeiro, Rio de Janeiro, Brazil; 2 DDS, PhD, Stomatology, Department of Diagnosis and Therapeutics, Dental School, State University of Rio de Janeiro, Rio de Janeiro, Brazil; 3 DDS, Post-graduation in Orthodontics, Dental School, State University of Rio de Janeiro, Rio de Janeiro, Brazil; 4 DDS, Post-graduation in Oral and Maxillofacial Surgery, Clementino Fraga Filho Hospital, Federal University of Rio de Janeiro, Rio de Janeiro, Brazil; 5 DDS, Pathology Department, Integra Cancer Center, Guatemala City, Guatemala; 6 DDS, PhD, Oral Pathology, Dental School, State University of Rio de Janeiro, Rio de Janeiro, Brazil

## Abstract

Peripheral odontogenic keratocyst (POKC) is a rare soft tissue entity showing the same histological characteristics of odontogenic keratocyst. Herein, we report two cases of POKC affecting the gingiva/alveolar mucosa. Case 1. A 43-year-old female was referred for evaluation of a painless well-defined nodular, sessile, non-tender swelling in the right maxillary buccal gingiva. No radiological alterations were observed. The patient was submitted to excisional biopsy and histological diagnosis was POKC. There were no signs of local recurrence after a 4-year follow-up. Case 2. A 63-year-old female was referred for evaluation of a painless well-defined nodular, sessile, yellowish swelling in the anterior mandibular alveolar mucosa. No radiological alterations were observed. The patient was submitted to excisional biopsy and histological diagnosis was POKC. Patient recovery was uneventful but she did not return for follow-up. POKC should be considered in the differential diagnosis of gingival cystic swellings and can be managed through conservative surgery.

** Key words:**Odontogenic keratocyst, peripheral, gingiva, alveolar mucosa.

## Introduction

Odontogenic keratocyst (OKC), a potentially aggressive odontogenic cyst, accounts for 12-14% of all odontogenic cysts and show a predilection for the posterior mandible (1,2). However, on rare occasions, OKC can affect only the soft tissues, with predilection for the gingival/alveolar mucosa. In these rare extraosseous cases, the term peripheral odontogenic keratocyst (POKC) has been applied (3). Therefore, we report two rare cases of POKC affecting the right maxillary gingiva and the anterior mandibular alveolar mucosa, both managed through a conservative surgical approach.

## Case Report

-Case 1

**Figure 1:**
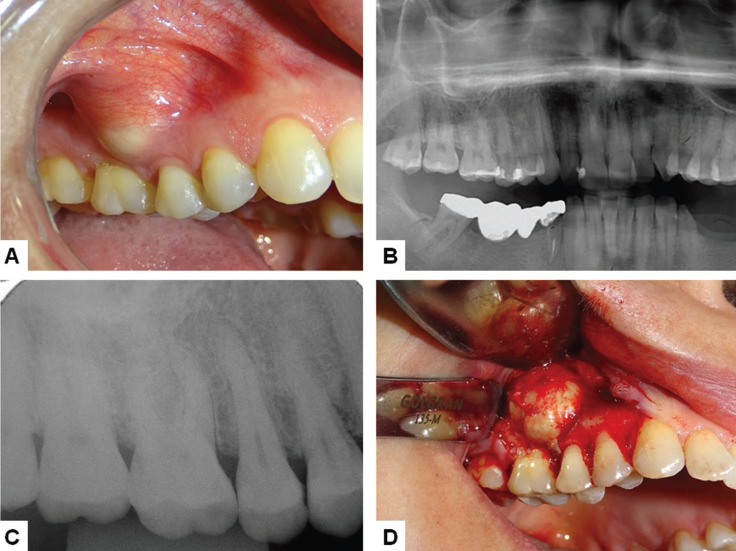
Case 1. A. Intraoral appearance of peripheral odontogenic keratocyst showing a well-defined nodular sessile swelling covered by normal oral mucosa in the upper right buccal gingiva between the second premolar and first molar. B. Panoramic radiograph showing no bone alterations in the right maxillary bone. C. Periapical radiograph showing no bone alterations in the area of the second premolar and first molar. D. Transoperative aspect showing a well-defined yellowish lesion superimposed to the cortical bone.

A 43-year-old woman was referred for evaluation of a 4-year lasting oral swelling in the right maxilla. Medical history was non-contributory and the patient denied any tobacco or alcohol consumption. Extraoral clinical examination showed no alterations. Intraoral clinical examination revealed the presence of a single painless well-defined nodular, sessile, non-tender swelling covered by normal oral mucosa, measuring 15 mm and located in the in the upper right buccal gingiva between the second premolar (#15) and first molar (#16) (Fig. 1A). Both teeth were vital and panoramic and periapical radiographs showed no alterations in the area (Fig. 1B,C). Clinical diagnosis was gingival cyst of the adult and an excisional biopsy was performed under local anesthesia (Fig. 1D). During the surgery a slight superficial bone resorption was observed, but there was no rupture of the cortical bone, confirming the location of the lesion exclusively in the soft tissues of the area.

**Figure 2:**
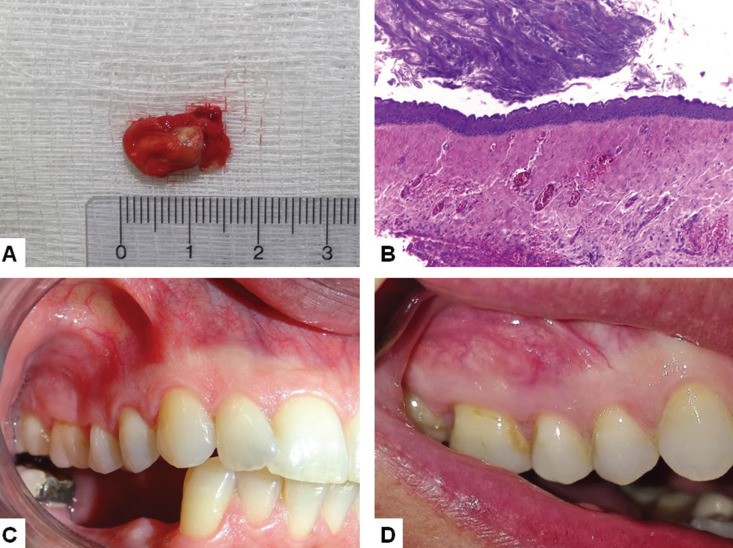
A. Gross image showing the yellowish content of the lesion. B. Histological features of the lesion showing a cystic cavity filled by keratin and lined by a parakeratinized stratified squamous epithelium with corrugated surface and polarized basal cell layer (HE, 100x). C. Postoperative aspect after a four-month follow-up, showing complete clinical repair in the area. D. Clinical aspect in a 4-year follow-up, showing no signs of local reccurence.

The specimen was immersed in 10% formaldehyde and sent for histological analysis (Fig. 2A). Hematoxylin and eosin stained 5 µm histological sections showed a fragment of oral mucosa covered by parakeratinized stratified squamous epithelium. In the adjacent connective tissue a cystic cavity filled by keratin and covered by a regular uniform parakeratinized stratified squamous epithelium with corrugated surface and a basal cell layer with a palisaded pattern was observed (Fig. 2B). The cystic cavity was located just beneath the surface epithelium and no bone fragments were observed. Due to the clinical, radiological, transoperatory and histological features a diagnosis of POKC was established. Patient recovery was uneventful and a four month follow-up showed complete clinical repair in the area (Fig. 2C). The patient remained in clinical and radiological follow-up and there are no signs of local recurrence in a four-year clinical follow-up (Fig. 2D).

-Case 2

**Figure 3:**
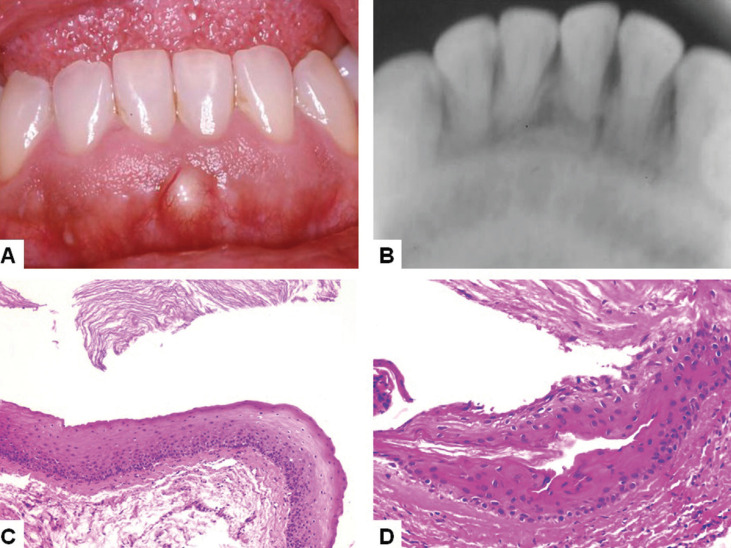
Case 2. A. Intraoral appearance of peripheral odontogenic keratocyst showing a well-defined sessile swelling covered by normal oral mucosa in the lower anterior alveolar mucosa between central incisors. B. Periapical radiograph showing no bone alterations in the area. C and D. Histological features of the lesion showing a cystic cavity filled by keratin and lined by a parakeratinized stratified squamous epithelium with corrugated surface and polarized basal cell layer (C - HE, 100x; D – HE, 200x).

A 63-year-old woman was referred for evaluation of an 18-month lasting oral swelling in the anterior mandibular alveolar mucosa. The patient reported controlled arterial hypertension. Extraoral clinical examination showed no alterations. Intraoral clinical examination revealed the presence of an asymptomatic single elevated lesion, covered by intact smooth surface mucosa, tense on palpation, with a yellowish coloration, measuring 10 mm in the lower alveolar mucosa between the central incisors (#31 and #41) (Fig. 3A). Both teeth were vital and a periapical radiograph showed no alterations in the area (Fig. 3B). Clinical diagnosis was gingival cyst of the adult and an excisional biopsy was performed under local anesthesia. During the surgery, cortical bone was intact and no central bone involvement was observed.

The specimen was immersed in 10% formaldehyde and sent for histological analysis. Hematoxylin and eosin stained 5 µm histological sections showed a cystic cavity filled by keratin and covered by a regular uniform parakeratinized stratified squamous epithelium with corrugated surface and a basal cell layer with a palisaded pattern (Fig. 3C,D). Due to the clinical, radiological, transoperatory and histological features a diagnosis of POKC was established. Patient recovery after one week was uneventful but she did not return for further follow-up.

We state that we have followed the Helsinki declaration and that written permission was obtained from both patients included in the present report.

## Discussion

OKC is a benign odontogenic cyst that shows some unique characteristics such as its typical histological pattern, potential for local infiltration and higher rates of local recurrence when compared with other odontogenic cysts (1,2). The condition has a slight predilection for males and most patients are in the second to fourth decades of life. The most common anatomical location for OKC is the posterior mandible and it presents as a well-defined unilocular or multilocular radiolucency. OKC usually appear as a solitary lesion but some sporadic cases can be associated to the nevoid basal cell carcinoma syndrome (Gorlin-Goltz syndrome) (1,2). 

In very rare instances OKC can present as an extraosseous soft tissue lesion with no involvement of the adjacent bone. POKC is expected to represent less than 0,5% of all OKCs, and 35 cases have been reported in the indexed English-language literature up to now (Table I) (3-24). Most cases (22 - 63% - including the present ones) affected the gingival/alveolar mucosa, with predilection for the upper buccal region from incisors to premolars (Table 1,  Table 1 cont.). Furthermore, POKC shows a slight male prevalence (1.1:1) and affect patients with a mean of 52 years (ranging from 16 to 83 years old); it usually presents as a soft tissue swelling measuring from 3 to 5 mm (3-24). The present cases have some interesting features as case 1 was located in the posterior maxillary gingiva and presented 15 mm in its largest diameter and case 2 affected a 63-year-old female.

**Table 1:**
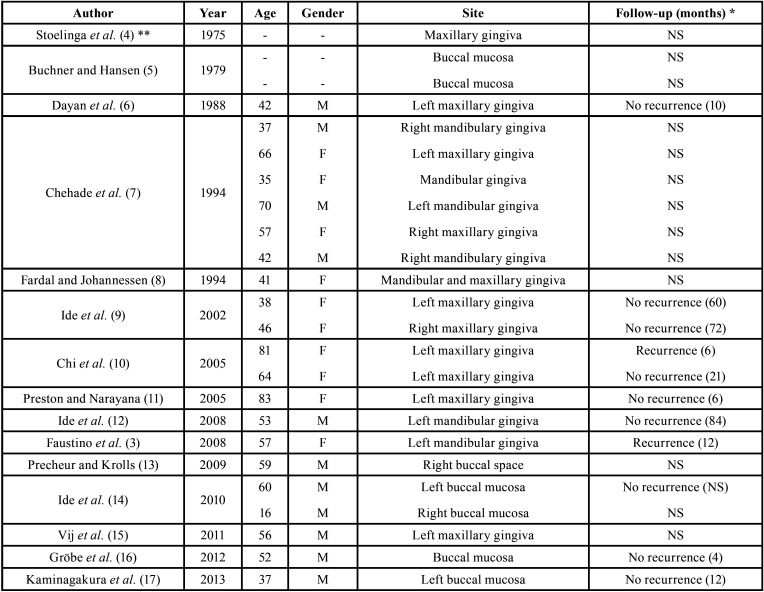
Peripheral odontogenic keratocysts reported in the English-language literature from 1975 to now.

**Table 1 cont.**
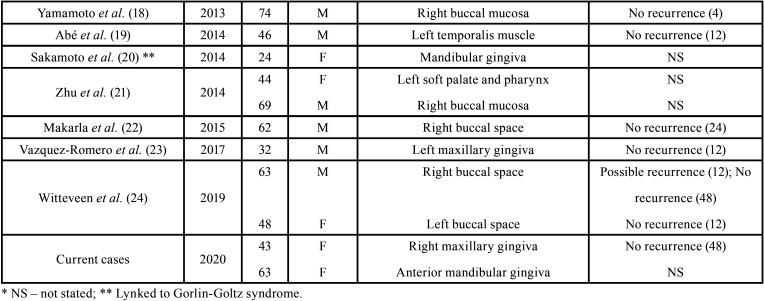
Peripheral odontogenic keratocysts reported in the English-language literature from 1975 to now.

Periapical and panoramic radiographs are essential in the diagnosis of any gengival swelling in order to exclude central intraosseous location of the lesion. To confirm the peripheral extraosseous location of the lesion no bone medullar destruction should be present, as shown in both present cases (21). In some cases, slight cortical saucer-shaped superficial resorption of the underlying bone can be seen, as shown in case 1 from this report, but integrity of the cortical bone should be observed.

As most POKC are located in the gingiva, differential diagnosis usually includes other odontogenic cysts that could affect this region, especially the gingival cyst of the adult and the peripheral calcifying odontogenic cyst. Both can produce a cup-shaped painless swelling filled by a bluish or bluish-gray fluid and superficial cortical bone resorption (9). The second location is the buccal mucosa and, in this site, cystic and cystic-solid salivary gland lesions should be considered in the clinical differential diagnosis. Histological analysis is the gold standard for POKC diagnosis. In the extraosseous location the same histological pattern described for conventional OKC should be present, as seen in the both present cases (2,3,6,9,12,15,21).

Due to the limited number of reported cases, there is still some controversy on the biological behavior of POKC. As conventional intraosseous OKC can be locally infiltrative, presenting a higher local recurrence rate when comparing to other cysts, many therapeutic options have been suggested for this condition (3,7,23). Nevertheless, it seems that POKC does not demonstrate the same aggressiveness and recurrence rates than central OKC. Therefore, its treatment will depend on the age of the patient, the location and size of the tumor, and whether it is a primary or recurrent tumor. Those characteristics justify the different therapeutic modalities for POKC reported in the literature, varying from conservative surgical removal - as performed in the present cases - to surgical removal with posterior curettage and a slight bone drilling of the area (4,7,12,23). Follow-up is available for 18 reported POKC and shows a low recurrence rate (3 cases – 17%), whereas the remaining 15 cases (83%) did not show any evidence of local recurrence in a mean of 27 months of follow-up (Table 1,  Table 1 cont.) (3,6,9-12,14,16-19,22-24). Case 1 has been followed-up for 48 months with no signs of local recurrence.

In conclusion, POKC is a rare condition that mostly affects adults and can affect both the posterior and anterior gingival/alveolar mucosa. It can clinically mimic other peripheral odontogenic cysts and should be managed by conservative surgical removal.
